# Risk Factors and Subtyping of Ischemic Stroke in Young Adults in the Indian Population

**DOI:** 10.7759/cureus.11388

**Published:** 2020-11-09

**Authors:** Vishali Moond, Kannu Bansal, Rohit Jain

**Affiliations:** 1 Internal Medicine, University College of Medical Sciences, New Delhi, IND; 2 Internal Medicine, All India Institute of Medical Sciences, New Delhi, IND; 3 Internal Medicine, Penn State Health Hershey Medical Center, Hershey, USA

**Keywords:** risk factors, ischemic stroke, stroke subtyping, young adults, indian population

## Abstract

Objective: To evaluate the risk factors and etiological subtyping of ischemic stroke in young adults in the Indian population.

Methods: This is a retrospective study of 160 patients, in the age group of 18 to 45 years with ischemic stroke, registered at a tertiary care hospital in Delhi, India between March 2014 and January 2018. Hypertension, diabetes mellitus, dyslipidemia, smoking, alcohol consumption, previous history of stroke, valvular heart disease, coronary artery disease (CAD), atrial fibrillation, family history, and migraine were considered as the identifiable risk factors. Stroke subtyping was done according to the Trial of Org 10172 in Acute Stroke Treatment (TOAST) criteria.

Results: The mean age of the patients was 36.2 years with 74% being males. Headache, vomiting, difficulty in speech, and hemiparesis were the common complaints at presentation. Common risk factors identified were hypertension (50%), prior stroke or transient ischemic attack (TIA; 32%), dyslipidemia (25%), family history of stroke (18%), and smoking (15%). The most common TOAST subtype was undetermined (64%), followed by other determined cause (ODC; 20%), and cardioembolism (15%).

Conclusion: There is a certain dissimilarity in the risk factors for ischemic stroke in young adults living in developing countries compared to those belonging to developed nations. Primary and secondary prevention targeted at the modifiable risk factors of ischemic stroke is necessary. Cerebral artery dissection, being a prevalent cause of ischemic stroke in young adults, should be carefully evaluated. A more appropriate stroke classification system specifically tailored for younger patients is needed.

## Introduction

Stroke is the leading cause of disability in adults and is the second most common cause of death worldwide. About two-thirds of stroke patients belong to developing countries [1]. In India and other developing countries, young adults constitute 15%-30% of all stroke patients as compared to 3%-8.5% in the West [2]. Young adults suffering from ischemic stroke have a larger bearing on the functioning of their family, society, and country as they are in their economically most productive period.

The causes of ischemic stroke in young adults are different than in the elderly. Also, careful evaluation of the stroke etiology is necessary for young adults to prevent recurrences.

Despite its significance, there is a scarcity of data on the risk factors and etiological subtyping of ischemic stroke in young adults in the Indian setting. We, through this study, aimed to fill this lacuna.

## Materials and methods

We retrospectively analyzed the data of 160 patients in the age group of 18 to 45 years with ischemic stroke registered at a tertiary care hospital in Delhi, India between March 2014 and January 2018. Ischemic stroke was defined as a sudden appearance of neurological signs and symptoms with imaging-confirmed cerebral infarction. Patients with intracranial hemorrhage, subarachnoid hemorrhage, and cerebral venous thrombosis were excluded.

Demographic, clinical and investigative data, and stroke risk factors were collected from the medical records. Investigations included hematological tests (complete blood count, blood sugar, serum cholesterol, serum triglyceride, prothrombin time, activated partial thromboplastin time), brain imaging (computerized tomography (CT), magnetic resonance imaging (MRI)), transthoracic echocardiography, and CT/MR angiography. 

Hypertension, diabetes mellitus, dyslipidemia, smoking, alcohol consumption, previous history of stroke, valvular heart disease, coronary artery disease (CAD), atrial fibrillation, family history, and migraine were considered as risk factors for stroke. Stroke subtyping was done according to the Trial of Org 10172 in Acute Stroke Treatment (TOAST) criteria [3].

Data were entered into Microsoft Excel, and frequency and proportion were calculated.

## Results

Demographics

The mean age of the patients was 36.2 years. Out of 160 patients, 118 (74%) were males and 42 (26%) were females. 

Symptoms and signs

The symptoms and signs at presentation are depicted in Table 1.

**Table 1:** Signs and symptoms at presentation

Signs and symptoms	Number of patients (n=160)
Affection of speech	96 (60%)
Right hemiparesis	89 (55%)
Headache	62 (39%)
Left hemiparesis	47 (30%)
Vomiting	40 (25%)
Loss of consciousness	24 (15%)
Seizure	16 (10%)
Quadriparesis	10 (6%)
Carotid bruit	4 (2.5%)

Risk factors

Risk factors identified were hypertension (50%), prior stroke or transient ischemic attack (TIA; 32%), dyslipidemia (25%), family history of stroke (18%), and smoking (15%) (Table 2).

**Table 2:** Identified risk factors

Risk factor	Number of patients (n=160)
Hypertension	80 (50%)
Prior history of stroke	51 (32%)
Dyslipidemia	41 (25%)
Family history	29 (18%)
Smoking	24 (15%)
Diabetes mellitus	19 (12%)
Alcohol	15 (10%)
Valvular heart disease	16 (10%)
Atrial fibrillation	13 (8%)
Coronary artery disease	11 (7%)
Migraine	5 (3%)

Stroke subtype

Stroke subtyping was done according to the TOAST criteria. The most common subtype was undetermined (64%), followed by other determined cause (ODC; 20%), and cardioembolism (15%) (Table 3).

**Table 3:** Stroke subtyping according to Trial of Org 10172 in Acute Stroke Treatment (TOAST) criteria

Stroke subtype	Number of patients (n=160)
Incomplete evaluation	157 (98%)
Undetermined- Total	102 (64%)
Other determined causes	32 (20%)
Cardio-embolic	24 (15%)
Large artery atherosclerosis	10 (6%)
Small artery disease	10 (6%)
More than two etiologies	5 (3%)

## Discussion

The majority (74%) of our patients were males. This is in contrast to the findings of the studies in the West [4,5]. However, several studies conducted in India have shown similar male predominance [6,7]. This might be the result of a sociocultural referral bias in India due to which males are more likely to seek treatment at a tertiary care center.

The presenting features such as headache, vomiting, difficulty in speech, and hemiparesis were similar to the previous studies conducted in India [6,8].

Hypertension was found to be the most common risk factor followed by prior stroke or TIA. Dyslipidemia and family history of stroke were the other leading risk factors for ischemic stroke. Although smoking and alcohol were found in 15% and 10% of the patients respectively, they were still less frequent compared to the studies conducted in the developed countries [9,10]. Prior stroke being a major risk factor might be because of lack of awareness for secondary prevention of stroke and hence, non-compliance to medications in patients. Aggressive primary and secondary prevention targeted at the modifiable risk factors of ischemic stroke is needed [7].

The most common stroke subtype was found to be undetermined (64%). This was significantly higher than the reported rate in the studies conducted in Western countries [11,12]. The possible reason behind this is the incomplete evaluation in the majority of our patients due to their unaffordability. Among ODC, cerebral artery dissection was reported to a prevalent cause of ischemic stroke in young adults and hence, its careful evaluation is necessary [13].

Cardioembolic stroke occurred in 15% of our patients. Valvular heart disease secondary to rheumatic heart disease (RHD) was its most common cause, as seen in the other studies conducted in the Indian setting [6,7,14]. In contrast to the studies conducted in the West, none of our patients were found to have patent foramen ovale, atrial septal aneurysm, or left atrial appendage thrombi [15]. This can be because we did not use transesophageal echocardiography (TEE) and hence, missed the diagnosis.

Five patients (3%) in the current study had migraine. Sacco et al. stated that migraine (especially with aura) should be considered as a risk factor for ischemic stroke and TIA [16]. Relation of stroke with preceding infection has also been hypothesized [17]. 

Alebeek et al., in 2018, proposed for an appropriate stroke classification system specifically tailored for younger patients. They stated that such a classification system would help to identify risk factors in patients otherwise classified as ‘‘unknown etiology’’ according to TOAST classification [18].

Limitations of our study were retrospective design, lack of complete evaluation, and a small number of patients.

## Conclusions

The current study highlights the dissimilarity between the developing and developed countries in the risk factors for ischemic stroke in young adults. History of prior stroke was found to be a major risk factor and hence, aggressive primary and secondary prevention targeted at the modifiable risk factors of ischemic stroke is needed. Cerebral artery dissection should be carefully evaluated in young patients with ischemic stroke. A more appropriate stroke classification system specifically tailored for younger patients is needed.
